# SIRT3 in post-myocardial infarction macrophage reprogramming: linking mitochondrial fitness to inflammation resolution and repair

**DOI:** 10.3389/fimmu.2026.1848626

**Published:** 2026-07-08

**Authors:** Xin Li, Xiaofei Luo, Zeng Zhang, Linya Tang, Xiudong Meng

**Affiliations:** 1 Qihuang College, Guizhou University of Traditional Chinese Medicine, Guiyang, China; 2 School of Basic Medicine, Guizhou University of Traditional Chinese Medicine, Guiyang, China

**Keywords:** efferocytosis, immunometabolism, inflammation resolution, macrophage reprogramming, mitochondrial fitness, myocardial infarction, NAD^+^, SIRT3

## Abstract

Myocardial infarction (MI) remains a leading cause of cardiovascular mortality worldwide. Despite significant advances in reperfusion strategies and pharmacotherapy, persistent inflammation and adverse ventricular remodeling continue to underlie poor long-term clinical outcomes. Macrophages serve as central orchestrators of post-MI healing, coordinating the clearance of necrotic debris, resolution of inflammation, remodeling of the extracellular matrix, and maturation of the fibrotic scar. However, the conventional M1/M2 dichotomy fails to fully capture the dynamic, phenotypically heterogeneous, and metabolically constrained macrophage states that emerge during infarct healing. In this review, we synthesize current evidence supporting a trajectory-based framework for macrophage reprogramming following MI and emphasize mitochondrial fitness as a critical determinant governing the transition from sustained inflammation to reparative resolution. We summarize key metabolic checkpoints regulating this functional shift—including glycolytic rewiring, tricarboxylic acid (TCA) cycle remodeling, mitochondrial reactive oxygen species (mtROS) accumulation, efferocytosis, oxidative phosphorylation (OXPHOS), fatty acid oxidation (FAO), and mitochondrial quality control. Furthermore, we advance the hypothesis that SIRT3—the principal mitochondrial NAD^+^-dependent deacetylase—may act as a central regulatory node linking mitochondrial protein acetylation to macrophage state transitions after MI. Specifically, we outline a staged dual-axis working model, generated from convergent but largely indirect evidence, in which the SOD2–mtROS axis is more closely linked to early nonresolving inflammation, whereas the PDHA1–metabolic flexibility axis may be more relevant to efferocytosis-associated reparative transition. We further highlight NAD^+^ availability as an upstream limiting factor that may constrain SIRT3 activity in macrophages under ischemic-inflammatory stress. Finally, we critically evaluate the current evidence hierarchy, human translatability, therapeutic strategies, and key translational challenges—emphasizing considerations of timing, cellular specificity, delivery modalities, and target engagement. Although macrophage-specific causal evidence in myocardial infarction (MI) remains sparse, this framework is intended as a mechanistically coherent and experimentally tractable working hypothesis to guide future investigations into macrophage immunometabolism and mitochondrial-targeted interventions in post-infarction cardiac repair. Accordingly, the proposed framework should be viewed as a testable working hypothesis rather than a settled causal model of macrophage fate control in MI.

## 1 Introduction

Myocardial infarction (MI) represents one of the most severe manifestations of acute coronary syndrome and remains a leading cause of cardiovascular mortality worldwide, imposing a substantial burden on public health (1). Although advances in reperfusion strategies and contemporary pharmacotherapy have significantly improved short-term survival, persistent inflammation and adverse ventricular remodeling continue to underlie late cardiovascular morbidity and mortality following MI (2). The pathobiology of post-MI healing unfolds in temporally distinct phases: ischemic injury predominates in the early phase, while inflammatory activation and tissue repair processes become increasingly dominant thereafter. Consequently, the ultimate clinical and structural outcome reflects the dynamic interplay among ischemic damage, inflammatory responses, and reparative mechanisms. Macrophages serve as central orchestrators of this continuum, mediating critical functions including clearance of necrotic debris, resolution of inflammation, extracellular matrix remodeling, and scar maturation (3). Following MI, cardiac macrophages originate from two principal sources: embryonically derived resident macrophages and monocyte-derived macrophages recruited from the circulation. These populations differ markedly in ontogeny, temporal kinetics, spatial localization, and functional specialization. They also undergo continuous, context-dependent shifts in response to evolving cues within the infarct microenvironment, progressing through stages of inflammatory activation, efferocytosis, reparative support, and chronic remodeling (4). Single-cell transcriptomic and genetic lineage-tracing studies further demonstrate that post-MI macrophages do not reside in static, discrete phenotypic states but instead exhibit spatiotemporal plasticity—dynamically transitioning across functional and metabolic states over time and location (5, 6). Accordingly, the conventional M1/M2 dichotomy is increasingly recognized as an oversimplified paradigm that fails to capture the full spectrum of macrophage heterogeneity, metabolic adaptability, and functional continuum observed in the infarcted myocardium (7).

In this review, we use the term ‘mitochondrial fitness’ to refer to an integrated mitochondrial state. This state includes respiratory competence, redox buffering capacity, substrate-handling ability, and quality-control integrity sufficient to support stage-appropriate macrophage functions during infarct healing. Research in immunometabolism has established that macrophage functional states are tightly coupled to mitochondrial fitness. Pro-inflammatory macrophages typically exhibit enhanced glycolysis, remodeling of the tricarboxylic acid (TCA) cycle, accumulation of immunoregulatory metabolites, and elevated mitochondrial reactive oxygen species (mtROS). In contrast, macrophages engaged in resolution and tissue repair rely predominantly on oxidative phosphorylation (OXPHOS), fatty acid oxidation (FAO), and robust mitochondrial quality control (8). Mitochondrial dysfunction in macrophages can directly compromise tissue repair, whereas targeted metabolic reprogramming can orchestrate transcriptional and epigenetic programs essential for recovery (9). Collectively, these findings indicate that mitochondrial function serves as a pivotal determinant governing the transition of macrophages from a pro-inflammatory state to a reparative phenotype.

SIRT3—the principal NAD^+^-dependent mitochondrial deacetylase—regulates key aspects of mitochondrial physiology, including protein acetylation, redox homeostasis, substrate utilization, and organelle integrity, and has been increasingly implicated in the pathogenesis of cardiovascular disease (10, 11). Building upon this evidence, we propose a staged, dual-axis conceptual framework: the SOD2–mtROS axis appears to predominate during persistent early inflammation, whereas the PDHA1–metabolic flexibility axis may be more closely associated with efferocytosis and the subsequent transition to repair. Within this framework, SIRT3 is considered a candidate mitochondrial regulatory node that may influence whether macrophages remain in a sustained inflammatory state or progress toward resolution and tissue repair. This integrative model may help reconcile existing observations and provide a mechanistic foundation for future studies focused on macrophage metabolism and function.

## 2 Macrophage diversity, trajectory, and functional evolution after MI

### 2.1 Resident and recruited macrophages in the infarcted heart

Macrophages infiltrating the infarcted heart originate predominantly from two sources: cardiac-resident macrophages and monocyte-derived macrophages recruited from the circulation. Under homeostatic conditions, resident macrophages maintain immune surveillance, support tissue homeostasis, and contribute to the integrity of local microenvironments (12). Following MI, circulating CCR2^+^ monocytes are rapidly mobilized to the injured myocardium and differentiate into macrophages that predominate during the early inflammatory phase. Importantly, these macrophage populations cannot be dichotomously categorized as either universally beneficial or detrimental. Resident macrophages exhibit context-dependent functional heterogeneity and are not uniformly protective; similarly, recruited macrophages display diverse functional roles and are not inherently deleterious. Instead, both populations execute distinct, stage-specific functions during injury response and tissue repair—functions dynamically shaped by hypoxia, local niche cues, and inflammatory mediators (13). Consequently, while cellular origin provides a foundational framework for understanding macrophage heterogeneity, it alone is insufficient to fully account for macrophage phenotype, functional specialization, or fate determination in the infarcted heart.

### 2.2 Temporal evolution and trajectory-based reprogramming after MI

**Figure 1:**
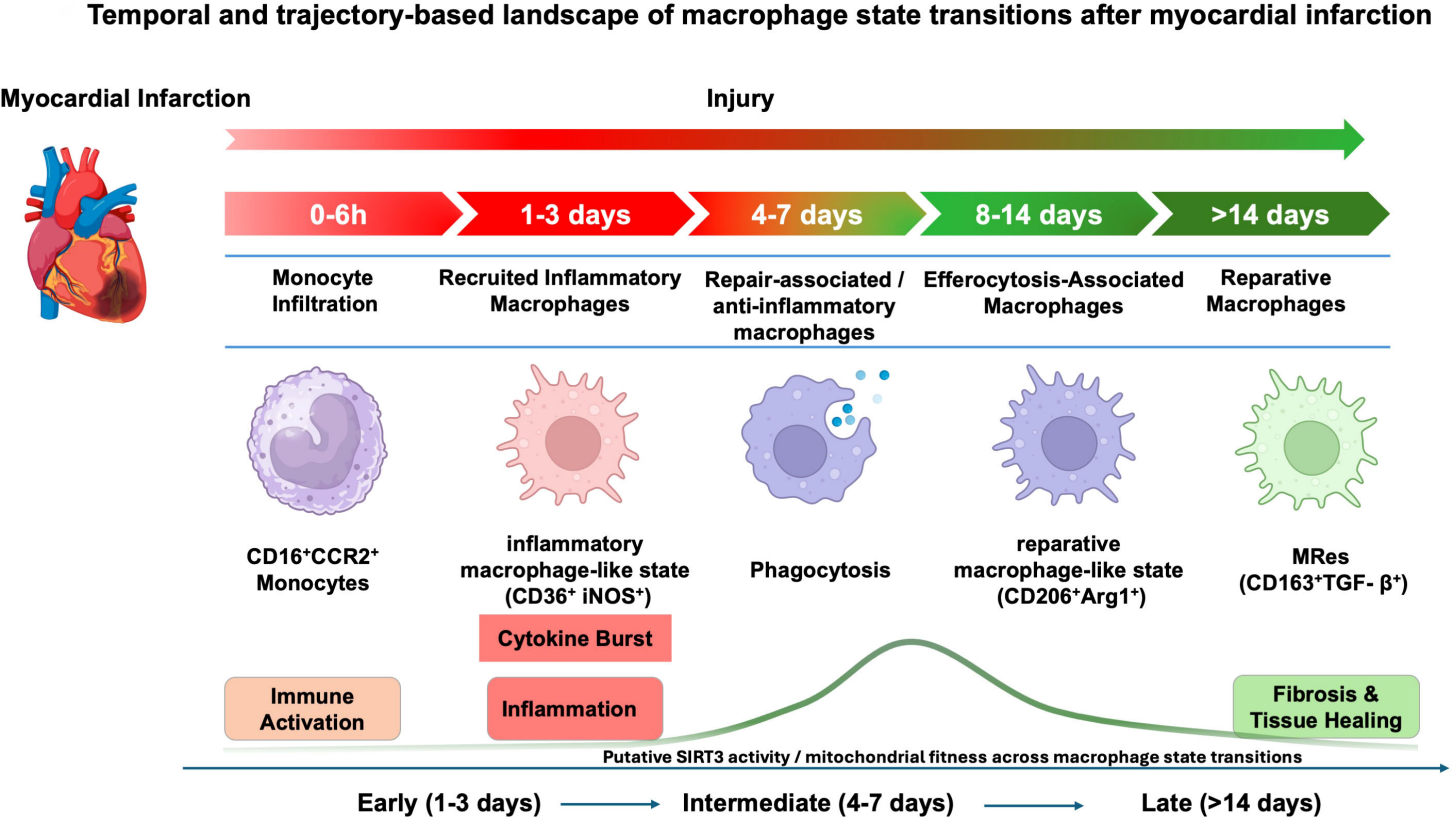
Temporal landscape of macrophage state transitions after myocardial infarction. Schematic overview of the dynamic, time-resolved program governing macrophage responses after myocardial infarction (MI). Following acute ischemic injury, circulating monocytes are rapidly recruited to the infarcted myocardium and differentiate into inflammatory macrophages that predominate during the early phase. These cells drive cytokine production, immune activation, and clearance of necrotic debris. Beginning around days 3–5 post-MI, macrophages progressively acquire efferocytosis-competent and pro-resolving phenotypes, enabling efficient clearance of apoptotic cells and facilitating the transition from inflammation to tissue repair. During the subsequent reparative phase, functionally specialized macrophage subsets promote angiogenesis, extracellular matrix remodeling, fibroblast activation and modulation, scar maturation, and overall tissue healing. In later stages, persistent macrophage populations continue to regulate fibrosis progression and contribute to chronic myocardial remodeling. This schematic underscores that post-MI macrophage biology cannot be adequately captured by a rigid M1/M2 dichotomy; instead, it is best conceptualized as a continuous, trajectory-based spectrum shaped by temporal cues, cellular origin, local microenvironmental signals, and functional demands. The annotation of putative SIRT3 activity/mitochondrial competence highlights the emerging concept that mitochondrial fitness may serve as a key regulator of macrophage state transitions across these temporally defined phases. These phases are shown as temporally ordered but partially overlapping states rather than discrete and mutually exclusive populations.

Following MI, macrophage responses unfold in a broadly sequential yet highly overlapping temporal program encompassing early inflammatory clearance, efferocytosis and resolution of inflammation, transition to a reparative phenotype, and ultimately, chronic remodeling. The initial inflammatory phase typically occurs within the first 1–3 days post-MI, characterized by rapid recruitment and accumulation of macrophages in the infarct zone. During this period, these cells exhibit heightened phagocytic capacity, elevated secretion of pro-inflammatory cytokines, and a metabolic shift toward glycolysis—supporting efficient clearance of cellular debris and amplifying innate immune signaling (14). A pivotal phenotypic transition ensues around days 3–5, marked by macrophage-mediated clearance of apoptotic neutrophils and the progressive acquisition of pro-resolving functional attributes. In the subsequent reparative phase, functionally distinct macrophage subsets increasingly contribute to key regenerative processes—including angiogenesis, modulation of fibroblast activation and proliferation, extracellular matrix (ECM) remodeling, and scar stabilization. At later stages (beyond day 7), macrophages persist in the healing myocardium, participating in scar maturation, sustaining low-grade inflammation, and maintaining fibrosis-associated signaling networks. Importantly, these phases are not temporally discrete; rather, they exhibit substantial spatiotemporal overlap and are continuously modulated by dynamic cues from the local microenvironment. Time-resolved single-cell transcriptomic studies further reveal that monocyte-derived macrophages undergo progressive functional diversification during infarct healing. Reparative subsets are strongly associated with efferocytosis, ECM regulation, and tissue repair at both the transcriptional and functional levels (5). (see **Figure 1**).

The continued reliance on the M1/M2 polarization paradigm risks oversimplifying macrophage biology following MI. This dichotomous model was largely derived from *in vitro* polarization systems and fails to capture the metabolically constrained, temporally dynamic, and multicellular microenvironment of the infarcted heart. *In vivo*, infarct-associated macrophages frequently co-express inflammatory, phagocytic, reparative, and extracellular matrix (ECM)-regulatory functions—rather than adopting discrete, mutually exclusive polarization states. A state-based, trajectory-aware framework thus provides a more physiologically accurate representation of post-MI macrophage biology. Post-MI macrophages are best conceptualized as heterogeneous populations undergoing dynamic reprogramming. These trajectories are shaped by four interdependent determinants: cellular origin, temporal context, functional demand, and metabolic state. Origin establishes the initial phenotypic bias; time dictates the dominant biological tasks; function encompasses inflammatory clearance, efferocytosis, reparative support, and ECM remodeling; and metabolism governs the capacity to execute these tasks (15). Efferocytosis is especially pivotal, as it couples metabolic adaptation with the production of pro-resolving mediators and facilitates the transition toward tissue repair. Accordingly, tracking the functional progression of macrophages—from inflammation through efferocytosis to repair—is biologically more informative than assigning static “M1” or “M2” labels. Consistent with this perspective, efficient efferocytosis, TREM2-dependent transcriptional programs, and macrophage metabolic remodeling have all been independently linked to improved infarct healing (16) (see **Table 1**).

**Table 1:** Macrophage states after MI: functional roles, metabolic features, and potential relevance to the proposed SIRT3 dual-axis working model.

Macrophage population/state	Origin	Predominant timing after MI	Functional role	Metabolic features	Representative markers	Key notes	Putative relevance to SIRT3 within the proposed working model	Putative SIRT3 axis	Evidence basis for SIRT3 assignment	Ref.
Resident cardiac macrophages	Embryonic-derived, self-maintained	Baseline; early phase retention	Immune surveillance, tissue homeostasis, early sensing	Oxidative metabolism, mitochondrial adaptability	TIMD4, LYVE1, FOLR2	Decline after injury but remain functionally relevant	May support mitochondrial resilience, but role remains unresolved	Context-dependent; currently unresolved	Conceptual alignment only	(3, 4, 17)
Recruited inflammatory macrophages	Monocyte-derived	Day 1–3	Necrotic clearance, cytokine production, inflammation amplification	Glycolysis-dominant, succinate accumulation, ↑mtROS, disrupted TCA	CCR2, IL1B, TNF	Necessary for early clearance but prone to persistence	Likely relevant to restraint of mtROS–inflammasome coupling	Predominantly aligned with the putative SOD2–mtROS axis	Indirect inference from inflammatory timing, metabolic phenotype, and non-MI macrophage studies	(11, 15, 18, 19)
Phagocytic/debris-clearing macrophages	Recruited ± resident	Early–intermediate	Debris clearance, high phagocytic load	High glycolytic flux with mitochondrial adaptation	Lysosomal markers	Overlaps with inflammatory states	May be relevant to mitochondrial stress adaptation during phagocytosis	Mixed/transitional (currently unresolved)	Conceptual alignment only	(12, 20)
Efferocytosis-associated macrophages	Recruited transition state	Day 3–5 (critical window)	Apoptotic cell clearance, inflammation resolution initiation	FAO activation, OXPHOS engagement, lipid handling, redox control	MERTK, CD36, TREM2	Critical checkpoint coupled to successful resolution	Likely supports metabolic adaptation and redox buffering during efferocytosis-associated transition	Predominantly aligned with the putative PDHA1–metabolic flexibility axis	Indirect MI relevance supported by macrophage metabolic studies outside MI	(7, 8, 21, 22)
Reparative macrophages	Reprogrammed recruited cells	Day 4–7+	Angiogenesis, fibroblast modulation, scar stabilization	OXPHOS, FAO, mitochondrial quality control, redox balance	IL-10, TGF-β, GPNMB	Not equivalent to M2; context-dependent	May act as permissive factor for oxidative reparative function	Likely linked to the PDHA1–metabolic flexibility axis	Mechanistically plausible but not directly established in MI macrophages	(5, 11, 23, 24)
Matrix-regulatory macrophages	Mixed origin	Late phase	ECM remodeling, fibrosis regulation	Less defined; likely oxidative/stress-adaptive	SPP1, SPARC	Involved in scar maturation	Role currently unclear; may be related to stress adaptation	Currently unresolved	Conceptual alignment only	(25, 26)
Chronic remodeling macrophages	Mixed, persistent populations	Chronic phase	Low-grade inflammation, fibrosis signaling	Metabolic dysregulation, persistent ROS	Remodeling-associated signatures	Poorly defined population	Likely phase-dependent and context-dependent	Phase-dependent and currently unresolved	Indirect inference only	(10, 27)

## 3 Immunometabolic checkpoints governing post-MI macrophage state transitions

**Figure 2:**
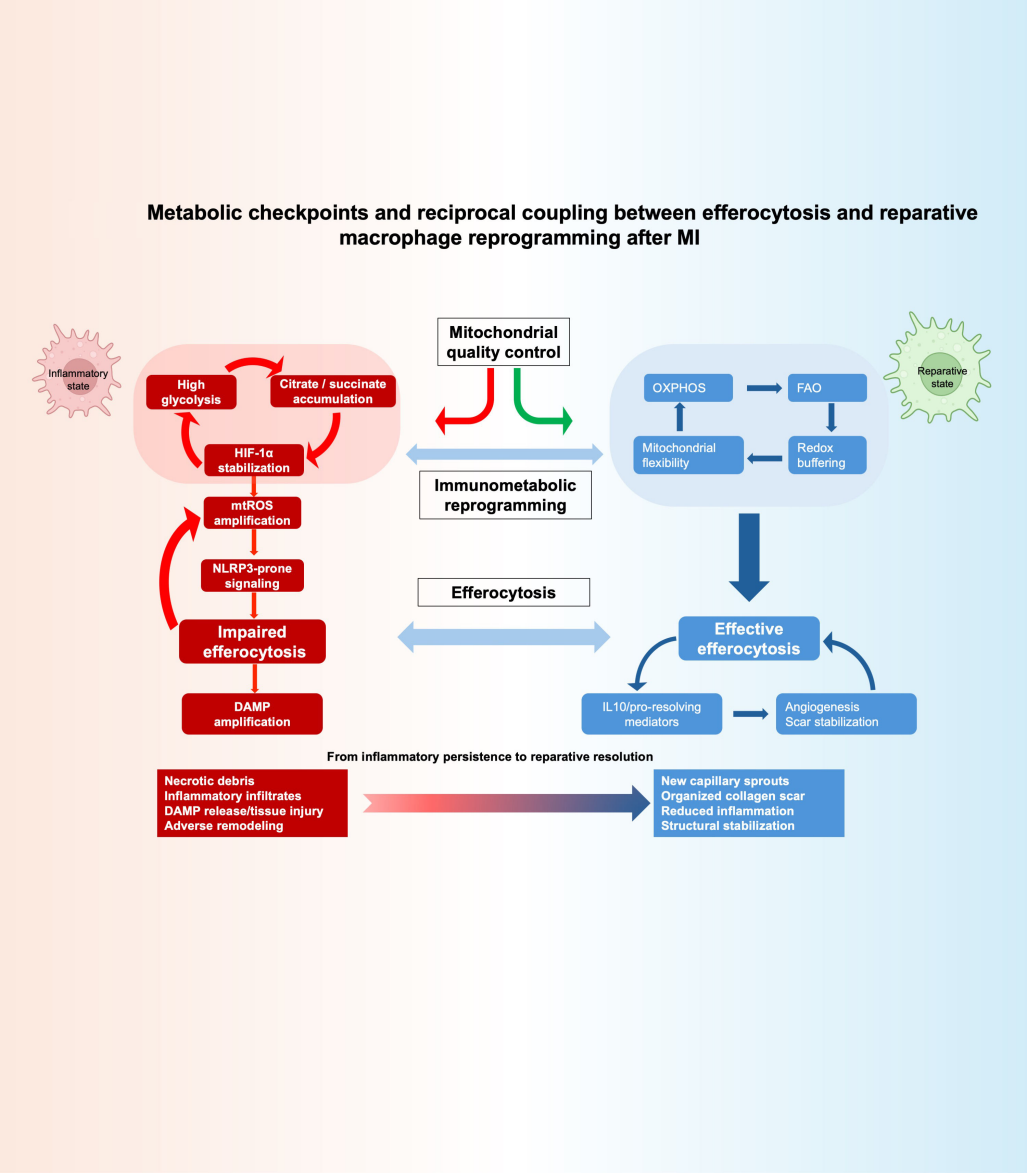
Metabolic checkpoints and reciprocal coupling between efferocytosis and reparative macrophage reprogramming after MI. Conceptual model illustrating key metabolic checkpoints associated with macrophage state transitions following myocardial infarction (MI). During the inflammatory phase, macrophages adopt a glycolysis-dominant metabolic program, characterized by remodeling of the tricarboxylic acid (TCA) cycle, accumulation of citrate and succinate, stabilization of hypoxia-inducible factor-1α (HIF-1α), amplification of mitochondrial reactive oxygen species (mtROS), and activation of inflammasome-prone signaling pathways—collectively associated with inflammatory persistence and amplification of danger-associated molecular patterns (DAMPs). In contrast, transition toward reparative states is associated with efficient efferocytosis, restoration of mitochondrial quality control, enhanced oxidative phosphorylation (OXPHOS) and fatty acid oxidation (FAO), increased mitochondrial flexibility, and strengthened redox buffering capacity. Importantly, efferocytosis is depicted not as a strictly unidirectional driver, but as a metabolically demanding checkpoint that is reciprocally coupled to reparative metabolic adaptation. This image summarizes macrophage immunometabolic reprogramming as a dynamic shift from inflammatory persistence toward reparative resolution and highlights mitochondrial function as a key permissive regulator of post-MI macrophage state transitions.

Macrophage state transitions following MI are orchestrated not only by extracellular inflammatory signals but also by intrinsic metabolic reprogramming. Consequently, cellular metabolism must be viewed as an active regulator—rather than a passive consequence—of macrophage fate decisions. Energy availability, metabolite-mediated signaling, redox homeostasis, and mitochondrial plasticity collectively determine the phenotypic spectrum macrophages can adopt. This occurs within the dynamically evolving infarct microenvironment. (see **Figure 2**).

### 3.1 Glycolysis, TCA cycle remodeling, and sustained inflammation

During the acute inflammatory phase, macrophages undergo rapid metabolic reprogramming toward aerobic glycolysis—a shift reminiscent of the Warburg effect. This adaptation enables rapid ATP production, provides biosynthetic precursors for macromolecule synthesis, and facilitates the expression of pro-inflammatory cytokines. Concurrently, the tricarboxylic acid (TCA) cycle is functionally remodeled: citrate accumulation diverts carbon flux toward fatty acid and itaconate biosynthesis, whereas succinate accumulation stabilizes hypoxia-inducible factor-1α (HIF-1α), thereby enhancing IL1B transcription. Moreover, succinate-driven reverse electron transport at mitochondrial complex I elevates mitochondrial reactive oxygen species (mtROS) production, which promotes NLRP3 inflammasome activation and contributes to the persistence of inflammation (28).

### 3.2 Oxidative metabolism, metabolic flexibility, and reparative states

Here, ‘metabolic flexibility’ refers to the capacity of macrophages to adapt substrate utilization and mitochondrial oxidative output in response to changing functional demands, including pyruvate oxidation, fatty acid utilization, and maintenance of respiratory competence under stress. In contrast to the pro-inflammatory phase, reparative macrophage states rely more heavily on oxidative phosphorylation (OXPHOS), fatty acid oxidation (FAO), and enhanced mitochondrial metabolic flexibility. Importantly, this metabolic profile should not be simplistically equated with canonical M2 polarization (29). Instead, it represents a functionally adaptive state in which mitochondria efficiently meet bioenergetic demands, minimize oxidative stress, and actively support tissue repair processes—including angiogenesis, extracellular matrix (ECM) remodeling, and the resolution of inflammation.

### 3.3 Efferocytosis, metabolite signaling, and mitochondrial quality control

In the context of MI, efferocytosis represents both a bioenergetic challenge and a pivotal signaling checkpoint. Upon engulfing apoptotic cardiomyocytes, macrophages must process substantial loads of lipids and cholesterol—imposing significant demands on mitochondrial capacity and function. Efficient efferocytosis enhances fatty acid oxidation (FAO), sustains mitochondrial respiratory activity, and upregulates the production of pro-resolving mediators—including interleukin-10 (IL-10). In contrast, impaired efferocytosis leads to secondary necrosis, sustained release of danger-associated molecular patterns (DAMPs), and consequent amplification of inflammatory responses (30). Thus, efferocytosis should be viewed not merely as a clearance mechanism, but as an energetically demanding checkpoint that is tightly coupled to reparative programming. A metabolic state permissive of repair may facilitate efficient efferocytosis, while successful efferocytosis can in turn reinforce pro-resolving and reparative macrophage functions. Recent studies further suggest that successful efferocytosis—particularly when coupled with TREM2-dependent signaling—is closely associated with, and may reinforce, reparative macrophage programs essential for infarct resolution and structural repair in the border zone (10, 22).

Metabolites serve dual roles: as energetic substrates and as potent signaling molecules that critically shape macrophage polarization, functional plasticity, and phenotypic commitment. For instance, succinate promotes pro-inflammatory activation by stabilizing hypoxia-inducible factor-1α (HIF-1α), whereas itaconate exerts anti-inflammatory effects via inhibition of succinate dehydrogenase (SDH) and concomitant activation of the NRF2-mediated antioxidant pathway. Lactate, too, functions as an epigenetic modulator—inducing histone lactylation to reprogram gene expression profiles. Within this intricate metabolic–signaling network, mitochondrial reactive oxygen species (mtROS) occupy a central regulatory node. Elevated mtROS exacerbates mitochondrial damage, triggers NLRP3 inflammasome assembly and activation, and—when mitochondrial quality control mechanisms (e.g., mitophagy and biogenesis) are compromised—reinforces a persistent pro-inflammatory macrophage phenotype (31).

Mitochondrial quality control—encompassing mitophagy, mitochondrial fission–fusion dynamics, and proteostatic regulation—is a critical regulator of macrophage functional plasticity following MI. Efficient clearance of damaged mitochondria and maintenance of a functionally adaptive mitochondrial network are essential for resolving inflammation and promoting tissue repair. In contrast, impaired mitochondrial quality control leads to persistent mitochondrial reactive oxygen species (mtROS) accumulation, aberrant inflammasome activation, defective efferocytosis, and failure to transition from pro-inflammatory to reparative macrophage phenotypes. Accumulating evidence indicates that macrophage-specific mitochondrial dysfunction compromises post-MI cardiac repair, whereas sustained macrophage dysregulation drives maladaptive inflammatory–fibrotic remodeling in the later stages (27).

### 3.4 Glutamine metabolism as an additional determinant of reparative programming

Glutamine metabolism has emerged as an additional component of macrophage immunometabolic remodeling that may influence polarization, inflammatory resolution, and tissue repair. Beyond serving as a carbon and nitrogen source, glutamine supports macrophage functional adaptation through glutaminolysis-derived generation of α-ketoglutarate (α-KG), replenishment of tricarboxylic acid cycle intermediates, and contribution to the hexosamine biosynthetic pathway. These processes are relevant to reparative macrophage programming because α-KG has been linked to transcriptional and epigenetic regulation of repair-associated phenotypes, whereas glutamine-derived UDP-GlcNAc may support glycosylation-dependent functions associated with macrophage activation state. In wound-healing settings, glutamine supplementation has been reported to promote CD206-associated M2-like polarization, increase IL-10 and TGF-β expression, and enhance angiogenic and matrix-remodeling responses, whereas inhibition of glutamine uptake or catabolism attenuates these effects (32).

Importantly, glutamine metabolism may also intersect directly with SIRT3-dependent macrophage regulation. Mechanistic work in IL-4-polarized macrophages has shown that activation of the SENP1–SIRT3 axis promotes glutaminolysis by deacetylating and activating GLUD1. This increases α-KG production and augments M2 polarization. In that setting, glutamine deprivation or GLS1 inhibition blunted reparative polarization, whereas cell-permeable α-KG partially rescued it, supporting a functional glutamine–α-KG dependence of SIRT3-linked macrophage reprogramming (33). In the context of MI, however, currently available evidence remains more inferential. Post-infarction macrophages exhibit dynamic changes in glutamine-related metabolites, glutamine metabolic gene expression, and glutamine utilization over the first week after injury, indicating that this pathway is engaged during infarct remodeling. Yet pharmacological glutamine supplementation or GLS1 inhibition had limited effects on bulk macrophage inflammatory markers and bioenergetics in that model, while more clearly affecting remote myocardial metabolism and left ventricular function (34). Accordingly, glutamine metabolism should be viewed as a biologically plausible contributor to macrophage state transitions and reparative adaptation after MI, and as a pathway that may intersect with SIRT3-dependent mitochondrial regulation, but its macrophage-specific causal role in infarct healing remains to be defined more directly.

## 4 SIRT3 as a mitochondrial regulatory node linking redox control, metabolic flexibility, and repair

SIRT3 as a compelling candidate mitochondrial regulatory node. These considerations raise a central question: Which upstream regulators can coordinately integrate mitochondrial metabolism, redox homeostasis, and inflammatory signaling in macrophages? SIRT3 stands out as a biologically plausible candidate. As the predominant NAD^+^-dependent mitochondrial deacetylase, SIRT3 directly governs the acetylation status of mitochondrial proteins, modulates reactive oxygen species (ROS) detoxification, regulates substrate utilization, and maintains organelle homeostasis.

### 4.1 Mitochondrial localization, NAD^+^ dependence, and the dual-axis framework

SIRT3 is predominantly localized to the mitochondrial matrix, and its enzymatic activity is strictly dependent on intramitochondrial NAD^+^ availability. This strategic subcellular positioning enables SIRT3 to sense and respond dynamically to fluctuations in mitochondrial energy status and redox state (35). Through site-specific deacetylation of key metabolic enzymes and antioxidant proteins—including core components of the tricarboxylic acid (TCA) cycle, fatty acid oxidation (FAO) machinery, and mitochondrial antioxidant systems—SIRT3 fine-tunes metabolic flux, ROS scavenging capacity, and overall mitochondrial integrity (36). In MI, NAD^+^ depletion and oxidative stress are hallmark features of mitochondrial dysfunction. Thus, from both spatial and mechanistic perspectives, SIRT3 occupies a pivotal node within the mitochondrial stress-response network.

Mechanistically, several SIRT3 substrates can be organized into a provisional staged dual-axis framework. This framework may help explain how mitochondrial lysine acetylation relates to distinct macrophage functional states. The first axis centers on SOD2-dependent regulation of mitochondrial reactive oxygen species (mtROS) and is predominantly associated with early pro-inflammatory activation. By deacetylating SOD2 at lysine 68, SIRT3 enhances its enzymatic activity, thereby reinforcing mitochondrial antioxidant capacity, limiting mtROS accumulation, and suppressing inflammasome activation—particularly via the NLRP3 pathway. Conversely, reduced SIRT3 activity leads to hyperacetylation and inactivation of SOD2, resulting in sustained mtROS signaling that can perpetuate inflammatory responses (37). The second axis revolves around PDHA1-dependent regulation of metabolic flexibility and is more closely linked to transitional and reparative macrophage phenotypes—especially during efferocytosis. Through site-specific deacetylation of PDHA1, SIRT3 promotes the mitochondrial oxidation of glycolysis-derived pyruvate. In doing so, it may facilitate a functional transition from a rigid, glycolysis-dominant metabolism toward a more flexible, oxidative metabolic program (38). This metabolic adaptability is critical when macrophages must simultaneously process lipid-laden apoptotic cells and maintain robust mitochondrial respiration to support clearance and tissue repair. Collectively, the SOD2–mtROS and PDHA1–metabolic flexibility axes may be conceptualized as a substrate-to-state regulatory framework. Within this framework, SIRT3 may exert stage-specific functions: during the initial inflammatory phase, it constrains excessive inflammation by preserving redox homeostasis; in later phases, it supports resolution and tissue repair by sustaining metabolic plasticity. Additional SIRT3 substrates—including IDH2 (involved in redox buffering) and LCAD (involved in fatty acid oxidation)—may also contribute to these processes; however, current evidence remains insufficient to assign them equivalent mechanistic weight relative to SOD2 and PDHA1 (39). At present, this substrate-to-state framework should be interpreted as a hypothesis-guided model for future macrophage-resolved validation rather than as definitive causal evidence. Its current value lies primarily in generating stage-specific and experimentally testable predictions, rather than in providing a settled mechanistic conclusion.

### 4.2 Mechanistically informative evidence outside MI and hierarchy of evidence in MI

Beyond MI, SIRT3 has been increasingly implicated in the regulation of macrophage immunometabolism across multiple pathological contexts—including infection, obesity, vascular inflammation, and atherosclerosis. In these settings, SIRT3 deficiency is consistently associated with elevated mitochondrial reactive oxygen species (mtROS), hyperactivation of the NLRP3 inflammasome, impaired metabolic flexibility, and exaggerated pro-inflammatory responses. In contrast, SIRT3 activation promotes oxidative phosphorylation and attenuates damage-associated molecular pattern (DAMP)-driven inflammatory amplification. Although these findings do not constitute direct evidence in MI models, they provide valuable biological context supporting a potential role for SIRT3 in post-MI macrophage biology (40). At the molecular level, studies in macrophage systems further demonstrate that SIRT3 coordinately regulates mitochondrial oxidative stress, inflammasome activity, and pyruvate dehydrogenase E1 alpha 1 (PDHA1)-dependent metabolic reprogramming (41, 42). The studies discussed in this section are cited primarily for mechanistic plausibility and should not be interpreted as direct evidence of macrophage-specific causality in MI. These studies are mechanistically informative but do not substitute for macrophage-specific *in vivo* evidence in MI. Accordingly, the relevance of this pathway to infarct macrophage biology remains inferential until directly validated in macrophage-specific MI models.

The existing literature should be interpreted according to the strength of causal inference. To date, no *in vivo* study has directly established a macrophage-specific function for SIRT3 in MI. Whole-heart analyses in MI or ischemia–reperfusion injury models—and studies lacking cell-type resolution—indicate that SIRT3 enhances mitochondrial function, mitigates oxidative stress, and suppresses injury-associated signaling pathways. Concurrently, mechanistic investigations in non-MI macrophage models reveal that SIRT3 modulates inflammatory signaling, mitochondrial quality control, and metabolic reprogramming. Collectively, these convergent lines of evidence position SIRT3 as a biologically plausible regulator of post-MI cardiac remodeling; however, they remain insufficient to establish a macrophage-intrinsic, causally defined role for SIRT3 in this setting (43). (see **Table 2**).

**Table 2:** Evidence hierarchy for SIRT3 in macrophage biology and post-MI remodeling.

Evidence tier	Study	Species/model	Disease/injury context	Cell specificity	Macrophage-related phenotype	MI/remodeling outcome	SIRT3-linked mechanism	Interpretive value for the proposed model	Major limitation	Implication for post-MI macrophage biology	Ref.
Tier 1: Direct macrophage-intrinsic causal evidence in MI	No eligible study identified among the currently reviewed full texts	—	Myocardial infarction/post-MI remodeling	—	—	—	—	Required but currently missing	A major knowledge gap: no macrophage-specific *in vivo* MI study directly establishes SIRT3 as a causal determinant of post-MI macrophage fate	This is the central missing layer needed to move the field from plausibility to macrophage-specific causality in MI	—
Tier 2: Indirect MI evidence	Cardioprotective effect of 19,20-epoxydocosapentaenoic acid (19,20-EDP) in ischaemic injury involves direct activation of mitochondrial sirtuin 3	Mouse ex vivo I/R, *in vivo* permanent LAD MI, and human LV fibers	Myocardial infarction/ischemia-reperfusion injury	Whole-heart/tissue-level	No direct macrophage phenotype resolved	Improved post-ischemic function, glucose oxidation, mitochondrial respiration, and cardiac efficiency	Direct activation of mitochondrial SIRT3; improved mitochondrial bioenergetics	Indirect but MI-relevant	Strong cardiac SIRT3 evidence, but no macrophage-specific readout	Supports the relevance of SIRT3-dependent mitochondrial adaptation in infarct tissue, but not whether macrophages are the key responder cell type	(43)
Tier 2: Indirect MI evidence	A Synthetic Epoxydocosapentaenoic Acid Analogue Ameliorates Cardiac Ischemia/Reperfusion Injury: The Involvement of the Sirtuin 3-NLRP3 Pathway	Isolated WT mouse hearts	Cardiac ischemia/reperfusion injury	Whole-heart/tissue-level	Inflammasome-related inflammation attenuated, but macrophage compartment not resolved	Improved postischemic recovery, ATP preservation, and reduced oxidative stress/NLRP3 activation	Preservation of mitochondrial SIRT3 activity and reduced MnSOD acetylation; SIRT3-NLRP3 linkage	Supportive for infarct-level plausibility	Cardiac-level inflammasome readout; no direct macrophage attribution	Supports the plausibility that SIRT3 may modulate inflammasome-prone biology in MI, a process highly relevant to inflammatory macrophages	(44)
Tier 2: Indirect MI evidence	Cardioprotective Effect of Licorice Extract Against Myocardial Ischemia and Ischemia/Reperfusion Injury via Blocking Cardiac Inflammation by Sirt3/NLRP3	Rat LAD ligation model plus H2O2-treated H9c2 cells	Myocardial ischemia and MI/R injury	Whole-heart/cardiomyocyte-centered	Reduced inflammatory mediators, but macrophage phenotype not directly resolved	Improved ECG/echo and pharmacodynamic outcomes; reduced inflammatory injury	Sirt3 upregulation inhibits NLRP3 inflammasome activation and restores metabolic homeostasis	Supportive for infarct-level plausibility	No macrophage-specific or myeloid-specific evidence	Further supports infarct-level compatibility between SIRT3 activity and reduced inflammatory amplification, but remains insufficient for macrophage-intrinsic interpretation	(45)
Tier 2: Indirect MI evidence	Cardiomyocyte-Specific JunD Overexpression Increases Infarct Size following Ischemia/Reperfusion Cardiac Injury by Downregulating Sirt3	Cardiomyocyte-specific JunD transgenic mice; constitutive Sirt3 knockout mice	Cardiac ischemia/reperfusion injury	Cardiomyocyte-specific/whole-heart	No macrophage-specific phenotype; systemic monocyte/macrophage chemoattractants were not altered	JunD overexpression enlarged infarcts; Sirt3 knockdown or deletion increased infarct size	JunD represses the Sirt3 promoter, causing mitochondrial swelling and cardiomyocyte death	Indirect but MI-relevant	Highly relevant to MI and SIRT3, but not macrophage-focused	Indicates that cell-type-specific SIRT3 effects matter in infarct biology and indirectly strengthens the rationale for analogous macrophage-specific testing	(46)
Tier 2: Indirect MI evidence	SIRT3 Transfection of Aged Human Bone Marrow-derived Mesenchymal Stem Cells Improves Cell Therapy-mediated Myocardial Repair	Aged human BM-MSCs transplanted into rat MI model	Myocardial infarction with cell therapy	Stem-cell-based/non-macrophage	No macrophage phenotype assessed	Improved graft survival, cardiac function, infarct size, fibrosis, and vascular density	SIRT3 enhances antioxidant defense via FOXO3a nuclear transfer, CAT, and MnSOD	Supportive for infarct-level plausibility	Transplantation study; does not resolve macrophage biology	Supports infarct-repair relevance of mitochondrial SIRT3 biology, but its implication for macrophage state transitions remains indirect	(47)
Tier 3: Direct macrophage evidence outside MI	Protective role of sirtuin3 against oxidative stress and NLRP3 inflammasome in cholesterol accumulation and foam cell formation of macrophages with ox-LDL-stimulation	WT and SIRT3-KO macrophages; ox-LDL stimulation	Atherosclerosis-related foam-cell model	Macrophage-specific *in vitro*	Reduced foam-cell formation, oxidative stress, mitochondrial dysfunction, and NLRP3 activation	Cardiovascularly relevant but outside MI	SIRT3 preserves mitochondrial function, restrains ROS and NLRP3, limits cholesterol accumulation	Mechanistically informative outside MI	Non-MI setting	Provides direct macrophage evidence that SIRT3 can couple mitochondrial integrity to inflammatory restraint, a logic potentially transferable to post-MI inflammatory macrophages	(48)
Tier 3: Direct macrophage evidence outside MI	SIRT3 (Sirtuin-3) Prevents Ang II (Angiotensin II)-Induced Macrophage Metabolic Switch Improving Perivascular Adipose Tissue Function	Myeloid SIRT3-deficient mice plus BMDMs	Ang II-induced PVAT inflammation/vascular remodeling	Myeloid-enriched with BMDM validation	SIRT3 loss drives glycolytic shift, IL-1β release, and NLRP3 activation	Worsened PVAT inflammation/fibrosis and vascular remodeling, but not MI	SIRT3 deacetylates PDHA1, restrains lactate accumulation and NLRP3-related inflammation	Mechanistically informative outside MI	Cardiovascularly relevant and mechanistically strong, but outside MI	One of the strongest pieces of substrate-level evidence supporting a substrate-to-state model highly relevant to inflammatory macrophages after MI	(49)
Tier 3: Direct macrophage evidence outside MI	SENP1-Sirt3 signaling promotes α-ketoglutarate production during M2 macrophage polarization	Macrophage polarization systems (IL-4-stimulated macrophages)	Alternative activation/reparative-like macrophage polarization	Macrophage-specific mechanistic evidence	Promotes M2-like polarization and reparative metabolic program	Provides mechanistic support for reparative macrophage biology, but not MI	SIRT3 deacetylates GLUD1, enhances glutaminolysis and α-ketoglutarate production	Mechanistically informative outside MI	Canonical polarization model; extrapolation to post-MI reparative macrophages is indirect	Supports the possibility that SIRT3 may facilitate oxidative reparative programs, but the relevance to true infarct reparative states needs validation	(33)
Tier 3: Direct macrophage evidence outside MI	Mitochondrial dysfunction caused by SIRT3 inhibition drives proinflammatory macrophage polarization in obesity	Macrophage-specific Sirt3 knockout mice, BMDMs, RAW264.7 cells	Obesity/adipose inflammation	Macrophage-specific plus *in vivo* metabolic inflammation	SIRT3 deficiency drives proinflammatory polarization and worsens inflammation	Systemic metabolic disease relevance; not MI	SIRT3 loss causes succinate dehydrogenase hyperacetylation, succinate accumulation, and KLF4 suppression	Mechanistically informative outside MI	Outside MI; obesity-specific context	Supports a mechanistic link between SIRT3 loss, metabolite dysregulation, and inflammatory macrophage persistence, which is conceptually relevant to infarct inflammation	(50)
Tier 3: Direct macrophage evidence outside MI	Sirtuin 3 Downregulation in Mycobacterium tuberculosis-Infected Macrophages Reprograms Mitochondrial Metabolism and Promotes Cell Death	Immortalized and primary murine macrophages; Sirt3-/- and LysMcreSirt3 mice	M. tuberculosis infection	Macrophage-specific plus systemic infection models	SIRT3 downregulation promotes mitochondrial metabolic derangement, ROS accumulation, and macrophage death	No MI remodeling outcome	SIRT3 regulates TCA/ETC homeostasis and redox balance under inflammatory stress	Mechanistically informative outside MI	Infectious context differs from sterile ischemic injury	Supports a broader principle that SIRT3 protects macrophage mitochondrial competence under inflammatory stress, but sterile infarct biology may differ substantially	(51)
Tier 3: Direct macrophage evidence outside MI	Small molecule-driven SIRT3-autophagy-mediated NLRP3 inflammasome inhibition ameliorates inflammatory crosstalk between macrophages and adipocytes	THP-1 macrophages, adipocyte co-culture, C57BL/6J mice	Metabolic inflammation/adipose tissue inflammation	Macrophage-centered with *in vivo* metabolic validation	SIRT3 activation restores autophagy, suppresses NLRP3 and inflammatory crosstalk	No MI remodeling outcome	SIRT3-AMPK-autophagy-mediated NLRP3 inhibition	Supportive but non-MI	Not MI-specific; obesity/adipose context	Supports the idea that SIRT3 may intersect with autophagy and inflammasome biology in macrophages, potentially relevant to infarct resolution	(52)
Tier 3: Direct macrophage evidence outside MI	SIRT3-mediated deacetylation of NLRC4 promotes inflammasome activation	Primary peritoneal macrophages, CRISPR iBMDMs, infection model	NLRC4 inflammasome activation/host defense	Macrophage-specific mechanistic evidence	SIRT3 deficiency impairs NLRC4 inflammasome activation and pyroptosis	No MI remodeling outcome	SIRT3 directly deacetylates NLRC4 to promote inflammasome assembly	Mechanistically informative outside MI	Mechanistically important but not cardiovascular and not NLRP3-centered	Important as a reminder that SIRT3 effects on inflammasomes may be context- and inflammasome-specific rather than uniformly suppressive	(53)
Tier 3: Direct macrophage evidence outside MI	Melatonin alleviates atherosclerosis by inhibiting pro-inflammatory differentiation of macrophages via regulating Sirt3-Drp1 mediated mitochondrial fission	ApoE-/- mice and RAW264.7 cells	Atherosclerosis	Macrophage-centered with *in vivo* vascular validation	Melatonin reduces proinflammatory differentiation and mitochondrial fragmentation in macrophages	Cardiovascular relevance outside MI	Sirt3-Drp1 axis limits mitochondrial fission and inflammatory macrophage differentiation	Supportive but non-MI	Atherosclerosis context; pharmacologic rather than genetic SIRT3 targeting	Supports a potential role for SIRT3 in mitochondrial dynamics, an axis that may also matter in infarct macrophage state transitions	(54)
Tier 3: Direct macrophage evidence outside MI	Adipocyte-expressed SIRT3 manipulates carnitine pool to orchestrate metabolic reprogramming and polarization of macrophages	Adipocyte-specific SIRT3 overexpression mice	Obesity/adipose tissue inflammation	Paracrine crosstalk model (adipocyte-intrinsic SIRT3, macrophage phenotype as downstream readout)	Reduced macrophage infiltration and proinflammatory polarization; enhanced anti-inflammatory polarization	No MI remodeling outcome	Adipocyte SIRT3 deacetylates CPT2, reshapes the L-carnitine pool, and secondarily reprograms macrophages	Supportive but non-MI	Macrophages are downstream responders rather than the manipulated cell type	Suggests that SIRT3-dependent immunometabolic effects may also emerge indirectly through microenvironmental reprogramming, relevant to multicellular infarct niches	(40)
Tier 4: Human/translational associative evidence	Nicotinamide Riboside Augments Human Macrophage Migration via SIRT3-Mediated Prostaglandin E2 Signaling	Human monocytes/macrophages with human NR supplementation	Human macrophage migration/wound-healing-related signaling	Human macrophage direct evidence	NR augments CCR7-dependent macrophage migration and wound-healing-like behavior	No MI remodeling outcome tested	SIRT3-dependent post-transcriptional COX-2/PGE2 signaling	Associative/translationally suggestive	Highly relevant to NAD^+^ support and translation, but not tested in MI	Supports translational interest in NAD^+^–SIRT3 manipulation in human macrophages, but not infarct-specific causality	(55)
Tier 4: Human/translational associative evidence	Untargeted metabolomics identified kynurenine as a predictive prognostic biomarker in acute myocardial infarction	Human STEMI cohort plus *in vitro* macrophage assays	Acute myocardial infarction/STEMI	Human associative with *in vitro* macrophage follow-up	Kynurenine promotes macrophage inflammation and oxidative stress	Worse 1-year MACCE after STEMI associated with higher plasma kynurenine	SIRT3-acSOD2-IL-1β signaling axis implicated	Associative and hypothesis-generating	Human data are correlative; no macrophage-specific *in vivo* causal validation	Provides tentative clinical relevance for a SIRT3-linked macrophage oxidative axis in MI, but remains correlative and hypothesis-generating	(37)

The table is intended to distinguish macrophage-intrinsic causal evidence in MI from indirect MI evidence and mechanistic macrophage evidence derived from non-MI contexts.

### 4.3 A staged dual-axis working model of SIRT3 action in post-MI macrophages

**Figure 3:**
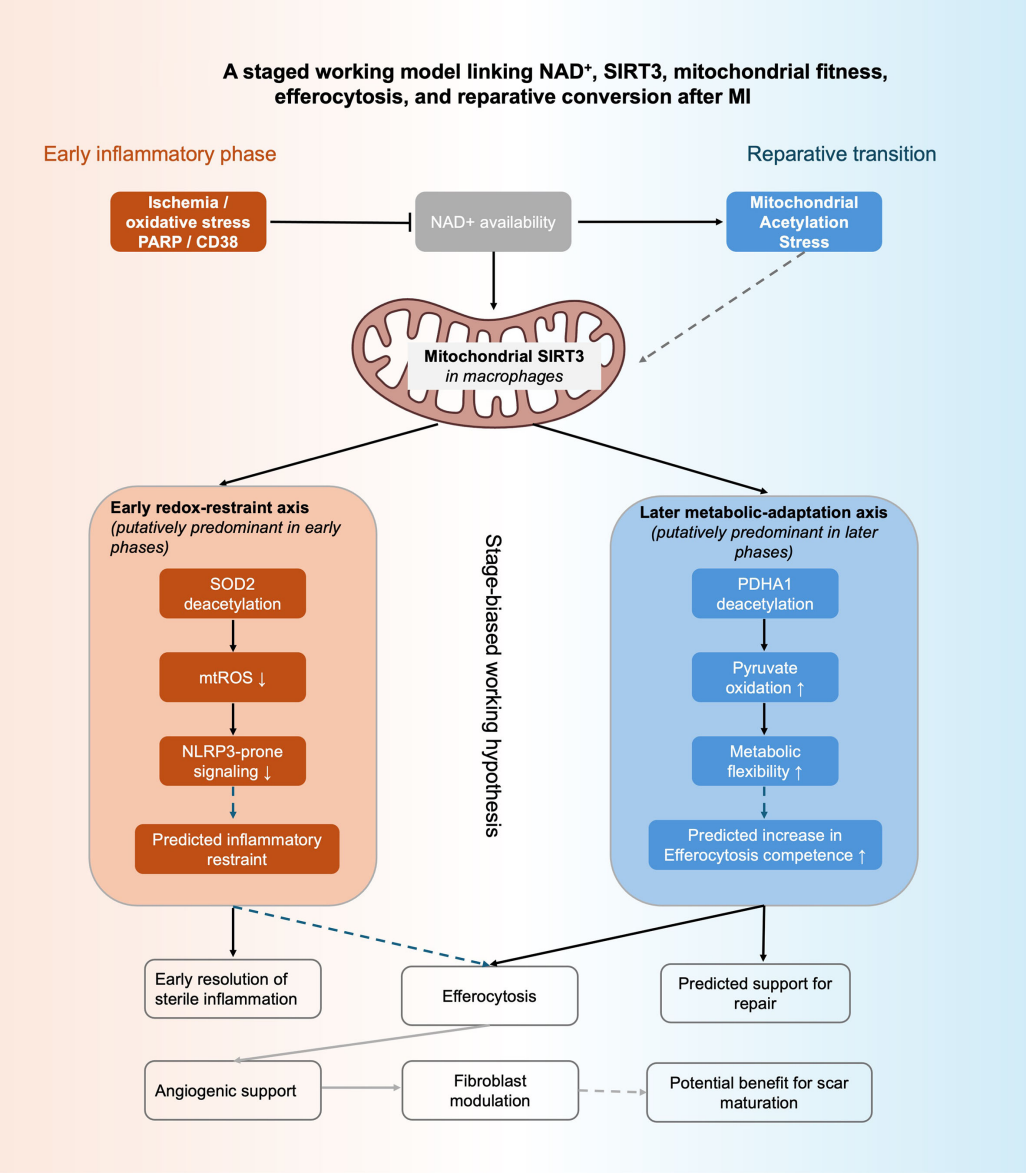
A staged working model linking NAD+, SIRT3, mitochondrial fitness, efferocytosis, and reparative conversion after MI. Proposed staged, dual-axis working model of SIRT3 function in post-myocardial infarction (MI) macrophages. Under ischemic and oxidative stress conditions, diminished NAD^+^ availability—combined with heightened acetylation stress—may impair mitochondrial SIRT3 activity. During the early inflammatory phase, SIRT3 is hypothesized to operate primarily through an early redox-restraint axis: by deacetylating superoxide dismutase 2 (SOD2), it curbs mitochondrial reactive oxygen species (mtROS) accumulation, dampens NLRP3 inflammasome–prone signaling, and thereby limits persistent sterile inflammation. As the response transitions toward tissue repair, SIRT3 is further proposed to engage a later metabolic-adaptation axis: deacetylation of pyruvate dehydrogenase E1 alpha subunit 1 (PDHA1) enhances pyruvate oxidation, improves metabolic flexibility, and augments efferocytic capacity. Through these putative, temporally coordinated, stage-biased actions, SIRT3 may help support timely resolution of early inflammation, facilitate the functional conversion of macrophages toward a reparative phenotype, and ultimately support angiogenesis, modulate fibroblast activity, promote scar maturation, and attenuate adverse cardiac remodeling. This schematic represents a mechanistically informed but still hypothesis-guided and empirically testable framework—rather than definitive, macrophage-specific causal evidence—in the context of MI.

Within this conceptual framework, SIRT3 modulates macrophage fate in a temporally regulated, stage-dependent manner by coordinately regulating redox homeostasis and metabolic flexibility. These two functional axes are unlikely to contribute equally across all phases of the post-MI response. Specifically, the SOD2–mitochondrial reactive oxygen species (mtROS) axis is predominantly implicated in sustaining early, maladaptive inflammation, whereas the PDHA1–metabolic flexibility axis appears more critical for facilitating the efferocytosis-driven transition toward a reparative macrophage phenotype (56). We present this model as a concise and experimentally testable working framework linking mitochondrial protein acetylation—particularly SIRT3-mediated deacetylation—to context-specific macrophage state transitions. At present, its value is primarily predictive, in that it generates stage-specific hypotheses for future macrophage-resolved testing in MI. Consistent with this, recent studies in MI models have demonstrated that discrete, stage-specific metabolic reprogramming programs are tightly coupled to tissue repair outcomes, underscoring the potential therapeutic relevance of the PDHA1-centered axis (57). (see **Figure 3**).

### 4.4 Functional implications for inflammation resolution, efferocytosis, repair, and multicellular remodeling

The central challenge in post-MI inflammation is not the initiation of the inflammatory response per se, but rather its timely resolution. Macrophages that fail to transition out of a pro-inflammatory state typically exhibit sustained glycolytic flux, impaired tricarboxylic acid (TCA) cycle function, and persistent mtROS generation. In this setting, the SOD2–mtROS axis exhibits the most direct and well-supported mechanistic link to pathological, nonresolving inflammation (58). By preserving mitochondrial antioxidant capacity—primarily through SOD2 activation—and supporting overall mitochondrial integrity and homeostasis, SIRT3 may attenuate the amplification of danger-associated molecular patterns (DAMPs), thereby curbing sustained macrophage activation.

Inflammasome activation—particularly via the mtROS–NLRP3 axis—represents a key mechanism that exacerbates myocardial injury following MI. Evidence from non–cell type–specific MI models and macrophage-focused studies in other pathological contexts indicates that SIRT3 may act upstream of this pathway by attenuating oxidative stress and maintaining mitochondrial integrity. Although macrophage-specific validation in the context of MI remains pending, the notion that SIRT3 modulates persistent inflammation and influences cellular susceptibility to inflammasome activation is strongly supported by mechanistic evidence (59).

Efferocytosis constitutes one of the most functionally informative interfaces linking SIRT3 to post-MI cardiac repair. Efficient recognition, internalization, and processing of apoptotic cells depend critically on intact mitochondrial respiration, coordinated lipid metabolism, and tightly regulated redox homeostasis (60). In this context, the PDHA1–metabolic flexibility axis provides a compelling mechanistic link, as it couples mitochondrial carbon flux to the energetic and biosynthetic demands inherent to efferocytosis. By preserving oxidative metabolic flexibility during this high-demand phase, SIRT3 may support the conditions under which efficient efferocytosis and transition toward a repair-supportive state become more likely. Conversely, efficient efferocytosis may itself stabilize a pro-resolving metabolic program, indicating a reciprocal rather than purely unidirectional relationship.

During the reparative phase, macrophages orchestrate key regenerative processes—including angiogenesis, fibroblast activation and phenotypic modulation, extracellular matrix (ECM) remodeling, and scar stabilization. These functions rely less on glycolytic metabolism—characteristic of sustained pro-inflammatory states—and more on enhanced oxidative capacity and metabolic flexibility. By augmenting mitochondrial efficiency and facilitating substrate oxidation—particularly of fatty acids and ketone bodies—SIRT3 may critically influence the functional programming of reparative macrophages. Nevertheless, current evidence remains insufficient to establish SIRT3 as a proximal, cell-autonomous regulator of reparative macrophage polarization *in vivo* (23, 25). Thus, current support for a reparative macrophage–intrinsic role of SIRT3 in MI remains suggestive rather than causally established.

Any assessment of SIRT3’s role in post-MI cardiac remodeling must account for the inherently multicellular nature of the injured myocardium. Macrophages modulate tissue repair not only via paracrine signaling—mediated by growth factors, cytokines, and extracellular vesicles—but also through direct physical and functional interactions with fibroblasts, endothelial cells, and cardiomyocytes. Concurrently, SIRT3 is expressed and functionally active in multiple cardiac cell types, including cardiomyocytes, fibroblasts, and endothelial cells, where it governs mitochondrial bioenergetics, redox homeostasis, and cellular stress resilience. This distinction has important interpretive consequences. Changes in SIRT3 activity in cardiomyocytes, fibroblasts, or endothelial cells may reshape the infarct microenvironment. This may occur through altered cell death burden, metabolite release, redox tone, matrix composition, angiogenic cues, and cytokine signaling. These changes can secondarily influence macrophage state transitions. Accordingly, tissue-level benefits attributed to the SIRT3 axis should not be assumed to reflect macrophage-intrinsic mechanisms unless supported by cell-type-specific evidence. Consequently, the tissue-level phenotypes attributed to SIRT3 activity likely arise from integrated intercellular crosstalk across this multicellular network, rather than from macrophage-intrinsic mechanisms alone.

## 5 NAD^+^ constraints, human relevance, and translational priorities

As an NAD^+^-dependent mitochondrial deacetylase, SIRT3 activity is governed not only by its expression level but—critically—by the local availability of mitochondrial NAD^+^. Consequently, the NAD^+^–SIRT3 axis assumes particular pathophysiological relevance within the myocardial infarct microenvironment. Ischemia, oxidative stress, DNA damage, and chronic inflammation collectively impair NAD^+^ biosynthesis, exacerbate NAD^+^ consumption (e.g., via hyperactivation of PARP and CD38), and disrupt mitochondrial NAD^+^ compartmentalization and homeostasis. These perturbations constrain SIRT3-mediated metabolic reprogramming and cytoprotective responses.

### 5.1 NAD^+^ availability as an upstream constraint on the SIRT3 axis

During MI, intracellular NAD^+^ depletion arises from multiple interrelated mechanisms: (i) compromised mitochondrial energy metabolism; (ii) excessive activation of poly (ADP-ribose) polymerase (PARP) secondary to ischemia-induced DNA damage; and (iii) upregulated activity of NAD^+^-consuming enzymes—most notably CD38. While tissue-level NAD^+^ depletion has been well documented—and observed across multiple non-macrophage cell types—the spatiotemporal dynamics of NAD^+^ flux specifically within infarct-associated macrophages remain poorly characterized. It remains unclear whether these immune cells undergo stage-dependent NAD^+^ loss and, if so, whether such loss is further constrained by subcellular compartmentalization (e.g., cytosolic versus mitochondrial pools) (61). Elucidating this question is essential for defining the functional contributions of macrophage NAD^+^ metabolism to post-MI inflammatory resolution and tissue repair.

Nicotinamide (NAM), nicotinamide riboside (NR), and nicotinamide mononucleotide (NMN) are promising metabolic interventions, as they elevate total cellular NAD^+^ levels and improve mitochondrial function in multiple preclinical models of injury. However, NAD^+^ is highly compartmentalized: cytosolic, nuclear, and mitochondrial pools are metabolically distinct and not freely exchangeable (46). Because SIRT3 is localized exclusively to the mitochondria, its enzymatic activity depends critically on the mitochondrial NAD^+^ pool—not on global or cytosolic NAD^+^ levels. Consequently, a systemic or tissue-level increase in total NAD^+^ does not guarantee restoration of mitochondrial NAD^+^ within infarct-associated macrophages. This limitation is further compounded by the multicellular nature of the infarcted heart, in which systemic NAD^+^ augmentation may alter cardiomyocyte, fibroblast, endothelial, and immune-cell metabolism in parallel. This limitation is especially pertinent when pharmacological studies attribute macrophage SIRT3 activation solely to measurements of total NAD^+^ or to downstream phenotypic outcomes. Although NR and other NAD^+^-boosting strategies have demonstrated cardioprotective effects in MI models, direct evidence demonstrating that these interventions selectively replenish mitochondrial NAD^+^ in macrophages remains lacking (55). Thus, the key unresolved question is not whether NAD^+^-supportive interventions elevate total tissue NAD^+^, but whether they restore the mitochondrial NAD^+^ pool sufficiently to support SIRT3-dependent regulation in infarct-associated macrophages.

Within the dual-axis framework, macrophage mitochondrial NAD^+^ availability may serve as an upstream constraint on both inflammatory restraint and reparative transition. In early inflammatory macrophages, insufficient mitochondrial NAD^+^ could impair SIRT3-mediated deacetylation of superoxide dismutase 2 (SOD2), thereby permitting pathological accumulation of mitochondrial reactive oxygen species (mtROS) (62). During the transition to, or within, reparative macrophage states, NAD^+^ deficiency may similarly compromise SIRT3-dependent deacetylation of pyruvate dehydrogenase E1 alpha 1 subunit (PDHA1), leading to diminished mitochondrial carbon flux and reduced metabolic flexibility—both of which are essential for efficient efferocytosis and subsequent tissue repair.

Several critical questions remain unresolved. First, systemic NAD^+^ elevation simultaneously affects cardiomyocytes, endothelial cells, fibroblasts, and multiple immune cell populations—thereby confounding the attribution of downstream phenotypes specifically to macrophage-intrinsic mechanisms. Second, temporal dynamics are likely decisive: the early ischemic–inflammatory phase and the subsequent repair–remodeling phase differ markedly in their biological demands and microenvironmental cues. Third, certain effects currently ascribed to macrophage-specific SIRT3 may instead reflect the activities of other NAD^+^-sensitive effectors—including SIRT1, PARP family members, or broader metabolic reprogramming. Accordingly, future studies should prioritize: (i) macrophage-specific metabolomics with subcellular resolution; (ii) genetically encoded biosensors for real-time, compartment-resolved monitoring of NAD^+^ dynamics; (iii) stable-isotope tracing to quantify precursor utilization and metabolic flux through the NAD^+^ biosynthetic and catabolic pathways; and (iv) stage-resolved characterization of NAMPT-, CD38-, and PARP-dependent NAD^+^ turnover in infarct-associated macrophages.

Although mechanistic insights in this field continue to rely heavily on animal models, emerging human studies corroborate the broader concept that MI is associated with profound immune reprogramming and metabolic remodeling. Analyses of peripheral blood, myocardial tissue biopsies, single-cell RNA sequencing (scRNA-seq), and spatial transcriptomics collectively demonstrate that macrophages infiltrating the human infarcted myocardium adopt a phenotypic continuum—ranging from pro-inflammatory to reparative states. Nevertheless, direct evidence linking SIRT3 activity or mitochondrial lysine acetylation to macrophage functional states in human MI remains scarce.

### 5.2 Human relevance of the proposed framework

Current human data consistently reveal post-MI expansion of inflammatory myeloid populations, dynamic transcriptional reprogramming across the monocyte–macrophage lineage, and the formation of spatially segregated immune niches within the infarcted myocardium. Collectively, these findings align with the state-continuum framework originally proposed in preclinical studies (63). However, they do not yet establish whether the NAD^+^–SIRT3 axis is functionally active in macrophages following human MI. An integrative analysis of publicly available scRNA-seq datasets and recent studies of human cardiac macrophages underscores the translational significance of TREM2-associated and reparative macrophage subsets. Yet, whether mitochondrial regulators—including SIRT3 or its downstream effectors—contribute mechanistically to the establishment or maintenance of these states remains experimentally untested (64).

### 5.3 Therapeutic strategies, timing, cell specificity, delivery, and target engagement

Therapeutic approaches targeting mitochondrial fitness and the NAD^+^–SIRT3 axis can be broadly classified into three categories: (i) interventions that directly or indirectly augment SIRT3-dependent mitochondrial function; (ii) NAD^+^-supportive strategies aimed at improving cellular cofactor availability; and (iii) platform-based approaches that modulate macrophages—or their local microenvironment—to enhance mitochondrial fitness, promote efferocytosis, or facilitate resolution of inflammation (65). These strategies vary considerably in their stage of preclinical and clinical development, as well as in the depth and rigor of mechanistic validation (66). To date, the most robust functional evidence in post-MI macrophages supports interventions that specifically enhance efferocytic capacity and macrophage-targeted immune–mitochondrial delivery systems. In contrast, direct pharmacological activation of SIRT3 remains conceptually compelling but lacks experimental confirmation of target engagement specifically in macrophages. Similarly, while systemic NAD^+^ supplementation shows promise in preclinical models, it has not yet been demonstrated to selectively elevate mitochondrial NAD^+^ levels within infarct-associated macrophages (67). Accordingly, any mechanistic link between systemic NAD^+^ supplementation and macrophage mitochondrial SIRT3 activation should currently be regarded as inferential rather than demonstrated. (see **Table 3**). From a near-term translational perspective, the most actionable strategies appear to be those that enhance efferocytosis, support metabolic flexibility during the day 3–5 transition window, and employ macrophage-targeted delivery platforms with measurable target-engagement readouts. By contrast, direct pharmacological activation of SIRT3 and systemic NAD^+^ supplementation remain conceptually attractive but currently less mature in terms of macrophage-specific mechanistic validation.

**Table 3:** Therapeutic strategies targeting mitochondrial fitness or the NAD^+^–SIRT3 axis: putative alignment with the proposed dual-axis working.

Strategy class	Representative approaches	Mechanistic rationale	Macrophage relevance	Preclinical MI evidence	Remodeling benefit	Evidence strength	Translational readiness	Putative primary SIRT3 axis targeted	Most critical translational bottleneck	Ref.
Direct SIRT3 activation	Gene delivery, 19,20-EDP, salvianolic acid B	May enhance mitochondrial deacetylation, redox control and metabolic adaptation	Largely inferred in macrophages	Moderate (non-macrophage-specific)	Improved function, reduced injury	Moderate	Low–moderate	Both axes (non-specific)	Lack of macrophage-specific targeting and macrophage-resolved target-engagement evidence	(43, 65, 67, 68)
NAD^+^ supplementation/NAD^+^-supportive interventions	NR, NMN, NAM, and CD38 inhibition	May improve whole-cell NAD^+^ availability and create conditions permissive for mitochondrial sirtuin activity; however, support for macrophage mitochondrial SIRT3 engagement in MI remains indirect.	Indirect and compartment-sensitive	Moderate	Reduced oxidative stress, improved function	Moderate	Moderate	Both axes (upstream constraint)	Uncertain elevation of mitochondrial NAD^+^ in target macrophages after systemic administration; compartmentalization of NAD^+^ pools; multicellular off-target or parallel effects	(69–72)
Mitochondrial antioxidants	ROS-responsive hydrogels, nanozymes	Reduce mtROS burden, limit inflammasome-prone signaling	Probable	Strong	Reduced infarct size, inflammation	Moderate–strong	Moderate–high	SOD2–mtROS axis	Attribution to SIRT3-dependent mechanisms remains unclear	(66, 73–76)
Mitophagy enhancement	PINK1/Parkin, PHB2 modulation	Improves mitochondrial quality, reduces damaged mitochondria	Indirect	Strong	Improved remodeling, reduced cell death	Moderate	Moderate	SOD2 axis (indirect support)	Macrophage-specific contribution unclear	(77–81)
Metabolic reprogramming	TREM2 axis, NR4A1–Drp1, hydrogels	Promotes oxidative metabolic adaptation and may support reparative transition	Direct	Strong	Improved repair and function	Strong	High (preclinical)	PDHA1–metabolic flexibility axis	Translation scalability and phase-matched implementation	(8, 82–86)
Efferocytosis enhancement	CD47-SIRPα blockade, apoptotic bodies	Improves apoptotic cell clearance and may reinforce resolution-associated reparative programming	Direct	Strong	Reduced fibrosis, improved healing	Strong	High	Putatively linked to the PDHA1–metabolic flexibility axis (functional output)	Timing precision	(87–89)
Targeted delivery systems	Nanoparticles, EVs, biomimetic carriers	Improves targeting and retention	Direct	Strong	Enhanced repair outcomes	Moderate–strong	Moderate–high	Axis-dependent (platform)	Manufacturing and reproducibility	(90–94)

**Figure 4:**
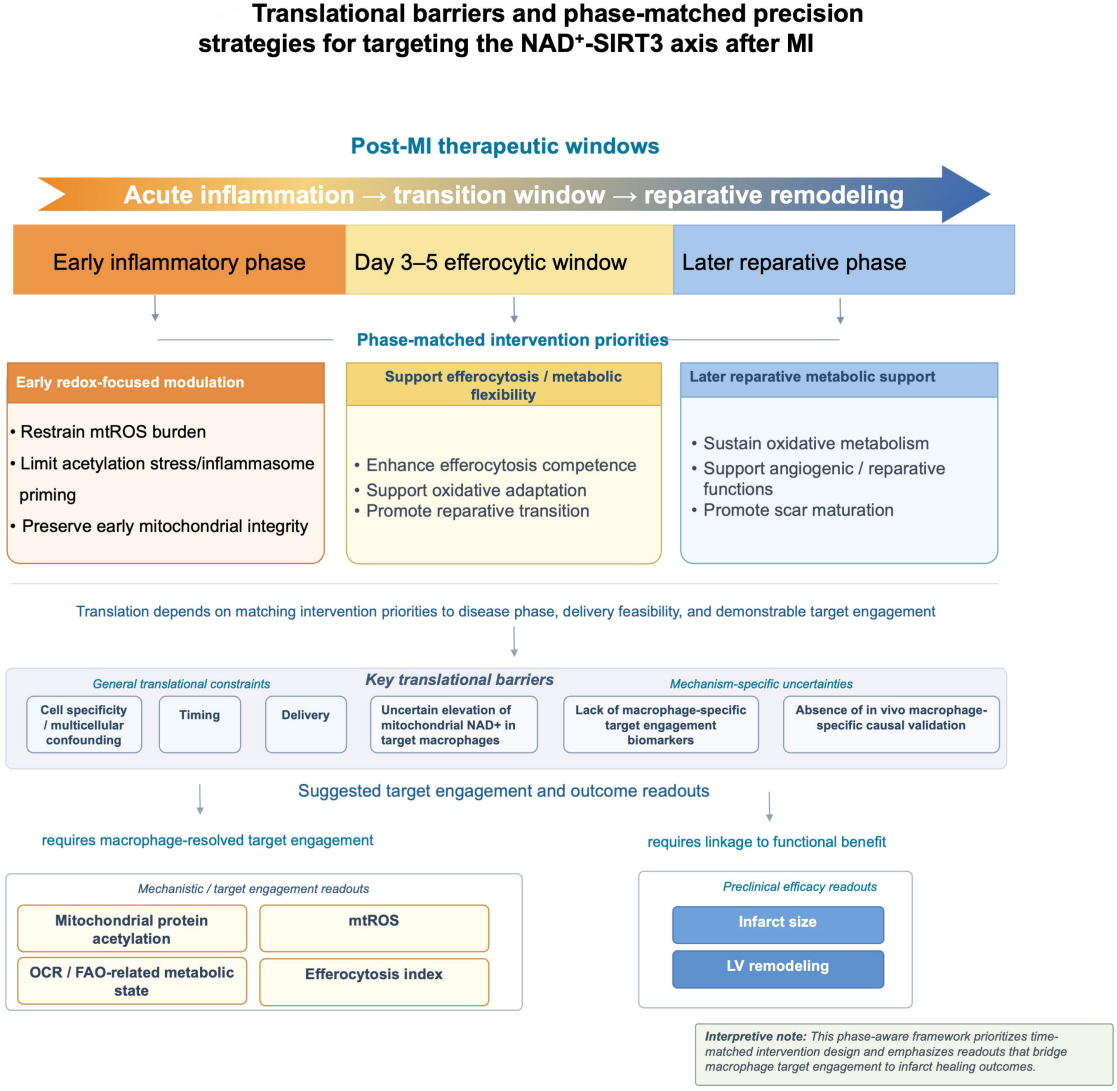
Translational barriers and precision strategies for targeting the NAD^+^-SIRT3 axis after MI. Phase-aware translational framework for therapeutic targeting of the NAD^+^-SIRT3 axis after myocardial infarction (MI). The schematic highlights three intervention windows: (i) an early redox-restraint phase aimed at limiting oxidative and inflammasome-prone stress while preserving mitochondrial integrity; (ii) an intermediate efferocytosis-support phase (days 3–5 post-MI) focused on enhancing efferocytosis, metabolic flexibility, and reparative transition; and (iii) a later reparative-metabolism phase intended to sustain oxidative metabolism and support scar maturation. The image also summarizes major translational barriers, including cell-type specificity, timing, delivery, uncertainty regarding mitochondrial NAD^+^ elevation in target macrophages, lack of macrophage-specific target-engagement biomarkers, and the absence of macrophage-specific causal validation *in vivo*. Representative readouts include mitochondrial protein acetylation, mtROS burden, oxidative metabolism, efferocytic capacity, infarct size, and left ventricular remodeling.

Successful clinical translation will critically depend on optimizing four interrelated parameters: timing of intervention, cell-type–specific targeting, efficient delivery to the intended site, and robust, mechanistically grounded evidence of target engagement. Early post-MI intervention must be approached with caution to avoid inadvertently suppressing essential inflammatory responses required for debris clearance and initiation of repair. In contrast, the phase of active efferocytosis—typically peaking between days 3 and 5 after MI—represents a more therapeutically favorable window for promoting resolution of inflammation and facilitating the transition from inflammation to tissue repair. Interventions administered later—beyond this peak efferocytic phase—may instead be better positioned to sustain reparative metabolism and mitigate adverse ventricular remodeling. Collectively, these temporal considerations point to three distinct, time-sensitive translational priorities: (i) early redox modulation to prevent the establishment of chronic, nonresolving inflammation; (ii) intermediate-stage support of efferocytosis and metabolic flexibility in phagocytic macrophages; and (iii) late-stage enhancement of reparative mitochondrial metabolism—specifically within defined, pro-reparative macrophage subsets. (see **Figure 4**).

An additional translational challenge is that systemic interventions directed at the NAD^+^–SIRT3 axis are unlikely to be macrophage-selective and may simultaneously affect multiple cardiac and immune cell populations, thereby complicating cell-specific interpretation. A major translational barrier remains the lack of validated, cell-type–specific biomarkers capable of reliably reporting target engagement within the relevant subcellular compartment (e.g., mitochondria). For the NAD^+^–SIRT3 axis, functionally informative readouts should include: (i) mitochondrial protein acetylation status (a direct downstream marker of SIRT3 deacetylase activity); (ii) mitochondrial reactive oxygen species (mtROS) burden; (iii) extent and directionality of macrophage metabolic reprogramming (e.g., shifts in glycolysis, oxidative phosphorylation, or fatty acid oxidation); and (iv) quantitative assessment of efferocytic capacity. These mechanistic endpoints should be interpreted in conjunction with established phenotypic outcomes—including infarct size, left ventricular ejection fraction, and diastolic/systolic function—to establish causal links between target modulation and functional benefit.

## 6 Key knowledge gaps, experimental priorities, and conclusions

Significant progress has been made in characterizing post-MI macrophage immunometabolism and SIRT3 biology. Nevertheless, several critical knowledge gaps persist and continue to impede both mechanistic understanding and translational advancement.

### 6.1 Key knowledge gaps and experimental priorities

The most pressing limitation is the absence of *in vivo* evidence from macrophage-specific genetic perturbation models demonstrating whether SIRT3 causally governs macrophage fate decisions following MI. This is not merely a technical shortfall; rather, it represents the central unresolved question in the field. Accordingly, future studies should prioritize rigorously controlled gain- and loss-of-function approaches using myeloid- or macrophage-specific genetic targeting—ideally integrated with well-validated MI models and high-resolution, state-resolved phenotyping of macrophage populations.

Recent single-cell studies have substantially enriched our descriptive understanding of macrophage heterogeneity after MI. However, investigations that jointly incorporate cellular lineage information and temporal resolution of state transitions remain scarce. A mechanistically grounded understanding of how distinct macrophage subsets dynamically navigate among inflammatory, efferocytic, and reparative functional states will require the integrative application of genetic lineage tracing, time-series single-cell multi-omics, spatial omics, and complementary functional validation (3, 95).

The proposed functional interplay among efferocytosis, mitochondrial fitness, and SIRT3 is conceptually compelling; however, direct experimental evidence remains sparse. Critical unresolved questions include: (i) whether SIRT3 directly modulates the uptake of apoptotic cells; (ii) whether efferocytosis triggers a temporally regulated program of mitochondrial protein acetylation; and (iii) whether mitochondrial NAD^+^ availability becomes rate-limiting during post-engulfment processing. Elucidating this tripartite interface represents one of the most pressing knowledge gaps within the current conceptual framework.

Future studies should integrate multi-omics approaches—including transcriptomics, metabolomics, and acetylomics—with cell-type–specific perturbation strategies. In addition to conventional inflammatory readouts, key functional endpoints should encompass: mitochondrial respiratory capacity; site-specific mitochondrial acetylation profiles; mitochondrial reactive oxygen species (mtROS) burden; quantitative assessment of efferocytic efficiency; and trajectory-resolved analyses of macrophage state transitions. Particularly informative experimental designs would employ macrophage-specific SIRT3 deletion or overexpression in clinically relevant MI models—such as permanent left anterior descending (LAD) coronary artery ligation and ischemia–reperfusion injury—combined with genetic lineage tracing, spatial transcriptomics, comprehensive mitochondrial acetylome profiling, and stage-specific metabolic phenotyping. Substrate-targeted rescue experiments—for instance, selective modulation of SOD2 activity or PDHA1 acetylation status—would provide a rigorous means to directly test the staged dual-axis model.

### 6.2 Conclusions

Post-MI inflammation is not merely a passive consequence of ischemic injury; rather, it is an active, dynamic, and highly orchestrated process that critically influences cardiac repair and long-term ventricular remodeling. Macrophages occupy a central role in this response, orchestrating multiple sequential functions—including clearance of cellular debris, resolution of inflammation, initiation of reparative signaling cascades, and maturation of the fibrotic scar. Accumulating evidence indicates that these temporally layered functions are profoundly regulated by mitochondrial fitness, thereby positioning immunometabolism as a pivotal determinant of post-MI macrophage biology.

Against this backdrop, SIRT3 represents a mechanistically plausible regulatory candidate. As the predominant mitochondrial NAD^+^-dependent deacetylase, SIRT3 modulates protein acetylation, redox homeostasis, metabolic substrate utilization, and overall mitochondrial integrity. Its principal significance lies in providing a unifying, biologically coherent, and experimentally tractable framework that links mitochondrial redox regulation and metabolic flexibility to macrophage phenotypic transitions following MI. The next critical step is to determine—using macrophage-specific genetic loss- and gain-of-function approaches—whether SIRT3 intrinsically and cell-autonomously governs key functional outcomes *in vivo*, including efferocytosis efficiency, transition to a reparative macrophage phenotype, and ultimately, structural and functional remodeling of the infarcted heart. Until such definitive causal evidence is obtained, the dual-axis model proposed herein should be regarded as a testable working hypothesis—intended to guide mechanistic investigation and inform translational prioritization—rather than an established conclusion.

## Data Availability

The original contributions presented in the study are included in the article/supplementary material. Further inquiries can be directed to the corresponding author.
